# SLC25A25-AS1 over-expression could be predicted the dismal prognosis and was related to the immune microenvironment in prostate cancer

**DOI:** 10.3389/fonc.2022.990247

**Published:** 2022-10-20

**Authors:** Ying-Ying Zhao, Qian-Ming Xiang, Jia-Li Chen, Li Zhang, Wei-Long Zheng, Di Ke, Rong-Shu Shi, Kong-Wu Yang

**Affiliations:** ^1^ Department of Radiology, Affiliated Hospital of Zunyi Medical University, Zunyi, China; ^2^ Department of Radiology, Fuqing City Hospital Affiliated to Fujian Medical University, Fuqing, China; ^3^ Department of General Surgery, The First Affiliated Hospital of Kunming Medical University, Kunming, China

**Keywords:** prostate cancer, SLC25A25-AS1, prognosis, biomarker, immune microenvironment

## Abstract

It has been established that long-chain coding RNA (lncRNA) SLC25A25-AS1 is associated with cancer progression. However, the roles and mechanisms of SLC25A25-AS1 in prostate cancer (PC) have not been reported in the literature. The present study explored the relationship between SLC25A25-AS1 expression and PC progression *via* comprehensive analysis. The pan-cancer expression of SLC25A25-AS1 was identified using data from The Cancer Genome Atlas (TCGA) database and tissue specimens from our hospital. The expression levels of SLC25A25-AS1 in various subgroups based on the clinical features were identified. The prognostic value of SLC25A25-AS1 and SLC25A25-AS1 co-expressed lncRNAs in PC patients was assessed by survival analysis and ROC analysis, and prognosis-related risk models of SLC25A25-AS1 were constructed. The relationship between SLC25A25-AS1 and the PC immune microenvironment was investigated using correlation analysis. SLC25A25-AS1 expression in PC was significantly increased and correlated with the T stage, clinical stage, Gleason score (GS), and dismal prognosis. SLC25A25-AS1 overexpression exhibited good performance in evaluating the prognosis of PC patients. The area under the curves (AUCs) of the 1-, 3-, and 5-year overall survival (OS) for SLC25A25-AS1 was 1, 0.876, and 0.749. Moreover, the AUCs for the 1-, 3-, and 5-year progress free interval (PFI) for SLC25A25-AS1 were 0.731, 0.701, and 0.718. SLC25A25-AS1 overexpression correlated with the infiltration of CD8 T cells, interstitial dendritic cells (IDC), macrophages and other cells. AC020558.2, ZNF32-AS2, AP4B1-AS1, AL355488.1, AC109460.3, SNHG1, C3orf35, LMNTD2-AS1, and AL365330.1 were significantly associated with SLC25A25-AS1 expression, and short OS and PFI in PC patients. The risk models of the SLC25A25-AS1-related lncRNAs were associated with a dismal prognosis in PC. Overall, SLC25A25-AS1 expression was increased in PC and related to the prognosis and PC immune microenvironment. The risk model of SLC25A25-AS1 have huge prospect for application as prognostic tools in PC.

## Introduction

Long non-coding RNAs (lncRNAs) are a class of RNAs longer than 200 nucleotides that can participate in the regulation of epigenetics, cell cycle, cell differentiation, and other functions (1–7). For instance, the lncRNA SNHG1 is significantly upregulated in oral cancer, and its inhibition can suppress oral cancer cell proliferation. Current evidence suggests that SNHG1 can regulate oral cancer growth by regulating the miR-421/HMGB2 signaling pathway (1). Moreover, overexpression of lncARSR could increase the resistance of colorectal cancer (CRC) cells to oxaliplatin *in vitro* and *in vivo*. Inhibition of lncARSR expression could reduce cell viability and promote cell apoptosis, and its upregulation could induce the tumorigenesis of CRC cells (3). LncRNA AP003469.4 was significantly increased in hepatocellular carcinoma (HCC) tissues. AP003469.4 overexpression was an influencing factor for dismal prognosis in HCC patients and was related to short overall survival (OS) and disease-free survival (DFS). Downregulation of AP003469.4 expression could delay the cell proliferation, cycle transition, invasion, and migration and promote cell apoptosis (6). This finding indicated that inhibiting or promoting the expression of lncRNAs could delay cancer progression.

Prostate cancer (PC) patients reportedly have a dismal prognosis and high mortality (8, 9). It was estimated that there will be more than 1,400,000 new cancer cases and more than 370,000 deaths in 2020 (9). The mainstay of treatment for PC patients is a comprehensive approach based on surgery. However, PC patients often have a dismal prognosis due to drug resistance and metastasis. An increasing body of evidence suggests that targeted therapy could improve the prognosis of cancer patients (10–13) and has gradually become a research hotspot. In recent years, lncRNA SLC25A25-AS1 has been associated with cancer progression (14, 15). The expression of SLC25A25-AS1 was significantly increased in the tissues and cells of non-small cell lung cancer (NSCLC). SLC25A25-AS1 overexpression was associated with shorter OS in patients with NSCLC. Importantly, SLC25A25-AS1 silencing could hinder cell proliferation, enhance the apoptosis rate of cells, and limit cell migration, invasion and tumor growth in NSCLC. Overall, SLC25A25-AS1 could exert a tumor-promoting effect by mediating the miR-195-5p/ITGA2 signaling pathway (14). Moreover, SLC25A25-AS1 has been reported to be significantly decreased in CRC tissues, and SLC25A25-AS1 overexpression could inhibit cell growth in CRC (15). No studies have hitherto reported the roles of SLC25A25-AS1 in PC progression. Therefore, this study sought to investigate the SLC25A25-AS1 expression in PC using a comprehensive analysis and the relationship between the overexpression level of SLC25A25-AS1 and the clinicopathological characteristics, diagnosis and prognosis of PC patients. The prognosis-related risk models of SLC25A25-AS1 were subsequently constructed. Finally, the relationship between SLC25A25-AS1 and the PC immune microenvironment was analyzed through correlation analysis to provide candidate targets for treating PC patients (**Figure S1**).

## Materials and methods

### The pan-cancer expression of SLC25A25-AS1

The expression data of TPM and FPKM types in pan-cancer tissues (730 cases of normal tissues and 10,363 cases of tumor tissues) were obtained from The Cancer Genome Atlas (TCGA) (https://portal.gdc.cancer.gov) database, and the expression data of TPM type in pan-cancer tissues (727 cases of normal tissues, and 9,807 cases of tumor tissues) were downloaded from the XENA-TCGA (http://xena.ucsc.edu/) database in March 2022. The mean expression levels of SLC25A25-AS1 in normal and cancer tissues were compared.

### Identification of the SLC25A25-AS1 expression in PC tissues

Cancer and normal tissues from patients (N=8) with a pathological diagnosis of PC treated in our hospital were analyzed. These PC patients provided informed consent for using their data. Our study was approved by the Ethics Committee of the Affiliated Hospital of Zunyi Medical University. SLC25A25-AS1 expression in PC patient tissues was detected by Reverse Transcription-Polymerase Chain Reaction (RT-PCR) technique. Total RNAs from PC patient tissues were collected according to standard specifications, and the reverse transcription process was performed after quantification. The PCR procedure was performed after primer addition, and the relative SLC25A25-AS1 expression level in PC tissues was calculated.

### Identification of the relationship between SLC25A25-AS1 expression and clinicopathological features and prognosis

The clinicopathological characteristics and prognostic data of PC patients were retrieved from TCGA database. The PC patients were grouped according to the clinicopathological characteristics, and different groups were compared to identify statistically significant differences. The expression data of SLC25A25-AS1 were analyzed, and PC patients were arranged into two groups according to the SLC25A25-AS expression value, and their prognostic values were compared by Kaplan-Meier (K-M) survival analysis. The relationship between the SLC25A25-AS1 expression and prognostic values in each subgroup based on the clinicopathological characteristics of PC patients were compared using K-M survival analysis.

### Assessment of the prognostic values of SLC25A25-AS1

Receiver operating characteristic (ROC) analysis is a widely acknowledged method used to assess the diagnostic accuracy of a particular test (16, 17). Over the years, ROC analysis has been used to assess the diagnostic value of molecules in cancer. AUC value’s closer to 1 were associated with higher diagnostic values. In the present study, the diagnostic values of SLC25A25-AS1 and SLC25A25-AS1 related lncRNAs expression levels in PC patients with the OS and progress free interval (PFI) at 1, 3 and 5 years were evaluated using ROC analysis.

### The relationship between SLC25A25-AS1 expression and the PC immune microenvironment

Immune cell scores of PC tissues were analyzed by the ssGSEA algorithm (18). The correlation analysis explored the relationship between the SLC25A25-AS1 expression and the PC immune infiltrating cells. The immune cell expression values were arranged from low to high, and the immune cells were divided into two (high- and low-expression) groups by the median values of the immune cells, and the SLC25A25-AS1 expression level in the high- and low-immune cell expression groups were explored.

### Identification of prognostic lncRNAs related to SLC25A25-AS1

The lncRNAs of OS and PFI in PC were analyzed by COX regression, and the significant OS- and PFI-related lncRNAs were screened using the criteria: P < 0.05 and P < 0.001. SLC25A25-AS1 co-expressed lncRNAs in PC tissues were acquired based on the screening criteria correlation coefficient r > 0.6 or r < -0.6 and P < 0.001. The intersected lncRNAs between the OS-related lncRNAs, PFI-related lncRNAs, and SLC25A25-AS1 co-expressed lncRNAs were visualized by a Venn plot. The intersected lncRNAs were defined as SLC25A25-AS1-related prognostic lncRNAs.

### Identification of the prognostic values of SLC25A25-AS1-related lncRNAs

The expression data of SLC25A25-AS1 related lncRNAs were retrieved from TCGA and matched and merged with the PC patient prognosis data. The expression data of SLC25A25-AS1 related lncRNAs were arranged, and the PC patients were grouped by the median expression value. K-M survival analysis was used to compare the relationship between SLC25A25-AS1-related lncRNAs and the OS and PFI of patients. patients. The value of SLC25A25-AS1-related lncRNAs AC020558.2, ZNF32-AS2, AP4B1-AS1, AL355488.1, AC109460.3, SNHG1, C3orf35, LMNTD2-AS1, and AL365330.1 for evaluating the OS and PFI at 1, 3, and 5 years was assessed by ROC analysis.

### Construction of the risk models associated with SLC25A25-AS1

The relationship between SLC25A25-AS1, AC020558.2, ZNF32-AS2, AP4B1-AS1, AL355488.1, AC109460.3, SNHG1, C3orf35, LMNTD2-AS1 and AL365330.1 expression with OS and PFI in PC patients was analyzed by LASSO algorithm. The risk models of SLC25A25-AS1-related lncRNAs were constructed. K-M survival analysis identified the prognosis roles of PC patients in high- and low-risk groups (19).

### Statistical analysis

The expression levels of SLC25A25-AS1 in the tissues and clinicopathological features of PC patients were explored using the Student’s t-test. K-M survival and ROC analysis were used to investigate the prognostic values of SLC25A25-AS1 and the co-expressed lncRNAs of SLC25A25-AS1. Correlation analysis was applied to understand the relationship between SLC25A25-AS1 expression and the PC immune microenvironment. A P-value < 0.05 was statistically significant.

## Results

### The pan-cancer expression of SLC25A25-AS1

**Figure 1:**
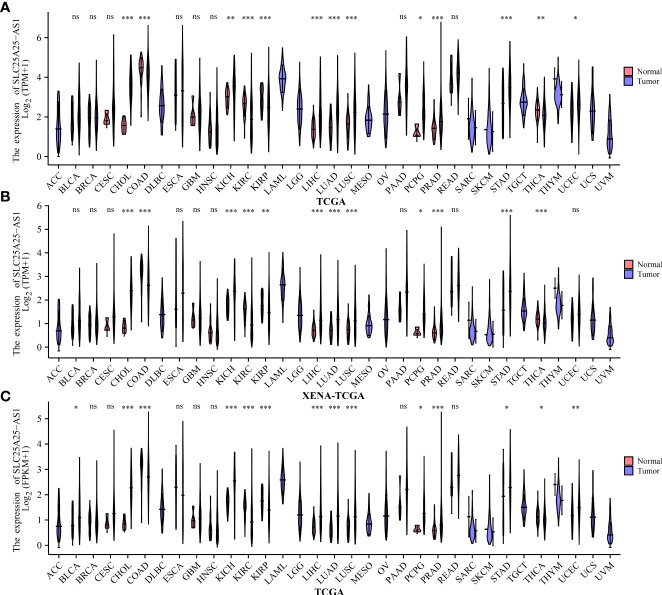
The expression values of SLC25A25-AS1 in pan-cancer tissues. **(A, B)** TPM type data of TCGA and XENA-TCGA databases; **(C)** FPKM type data of TCGA database. ns, not statistically significant; *, P < 0.05; **, P < 0.01; ***, P < 0.001.

We found that SLC25A25-AS1 was significantly overexpressed in CHOL, KICH, LIHC, LUAD, LUSC, PCPG, PC, STAD and UCEC tissues and downregulated in COAD, KIRC, KIRP and THCA tissues using the TPM type data from TCGA and XENA-TCGA database (**Figures 1A, 1B**). It was found that SLC25A25-AS1 was significantly overexpressed in CHOL, KICH, LIHC, LUAD, LUSC, PCPG, PC, STAD, and UCEC tissues and downregulated in COAD, KIRC, KIRP and THCA tissues based on FPKM data from TCGA database (**Figure 1C**).

### SLC25A25-AS1 overexpression is significantly related to the T stage, clinical stage, GS, and dismal prognosis in PC patients

**Figure 2:**
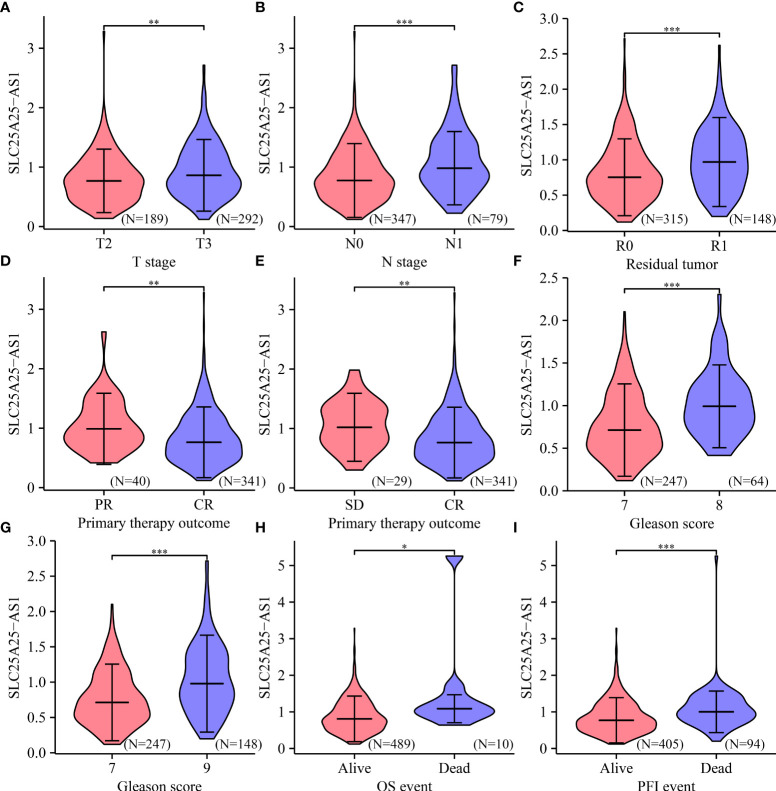
The expression levels of SLC25A25-AS1 in clinicopathological features of PC patients. Differences in OS and PFI between **(A)** T2 vs. T3; **(B)** N0 vs. N1; **(C)** R0 vs. R1; **(D)** PR vs. CR; **(E)** SD vs. CR; **(F)** GS7 vs. GS8; **(G)** GS7 vs. GS9; **(H, I)** Alive vs. Dead. GS, Gleason score; OS, overall survival; PFI, progress free interval; *, P < 0.05; **,P < 0.01; ***, P < 0.001.

**Figure 3:**
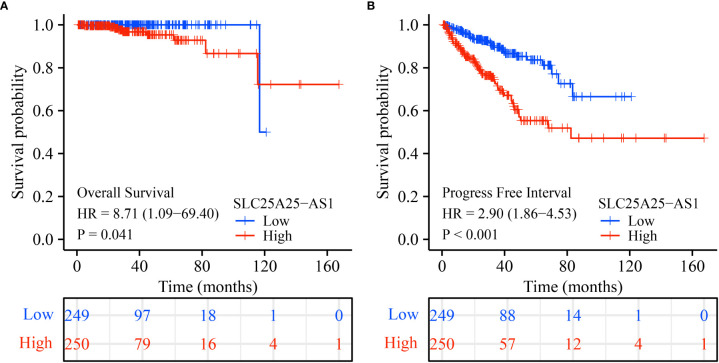
The poor prognosis in PC patients with SLC25A25-AS1 overexpression. **(A)** OS; **(B)** PFI. PC, prostate cancer; OS, overall survival; PFI, progress free interval.

Analysis of our clinical samples consistently showed that SLC25A25-AS1 expression was significantly up-regulated in most PC patients (**Figure S2**). SLC25A25-AS1 expression was significantly elevated in T3 stage PC patients than in T2 stage patients (**Figure 2A**), and was significantly increased in N1 stage PC patients compared with N0 stage PC patients (**Figure 2B**). SLC25A25-AS1 expression was significantly increased in PC patients with positive surgical margins (R1) compared with PC patients negative for residual tumor (R0) (**Figure 2C**). SLC25A25-AS1 expression was significantly elevated in partial remission (PR) patients compared with complete remission (CR) patients (**Figure 2D**). Moreover, SLC25A25-AS1 expression was significantly elevated in PC patients with stable disease compared with the PC patients in CR (**Figure 2E**), and SLC25A25-AS1 was significantly increased in PC patients with Gleason Scores (GS) of 8 and 9 compared to PC patients with GS of 7 (**Figures 2F, G**). In terms of OS and PFI, higher SLC25A25-AS1 expression correlated with a poorer prognosis (**Figures 2H, I**). K-M survival analysis showed a significant correlation between SLC25A25-AS1 overexpression and a short OS and PFI (**Figure 3**).

### SLC25A25-AS1 overexpression has prognostic values in diagnosing PC patients

**Figure 4:**
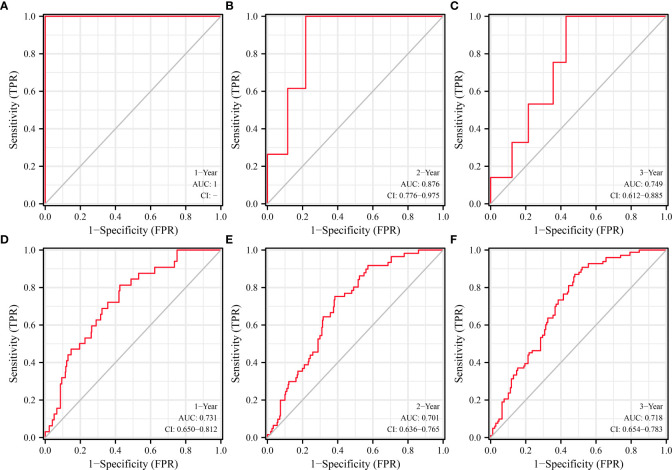
SLC25A25-AS1 overexpression had significant value in predicting the prognosis of PC patients. **(A–C)** OS; **(D–F)** PFI. PC, prostate cancer; OS, overall survival; PFI, progress free interval.

ROC analysis found that SLC25A25-AS1 overexpression has significant value in evaluating the prognosis of PC patients. The AUC of SLC25A25-AS1 for predicting OS at 1, 2 and 3 years was 1 (**Figure 4A**), 0.876 (**Figure 4B**) and 0.749 (**Figure 4C**), respectively. The AUC of SLC25A25-AS1 for PFI at 1, 2 and 3 years was 0.731 (**Figure 4D**), 0.701 (**Figure 4E**) and 0.718 (**Figure 4F**), respectively.

### SLC25A25-AS1 overexpression is associated with a dismal prognosis in subgroups of PC patients

**Figure 5:**
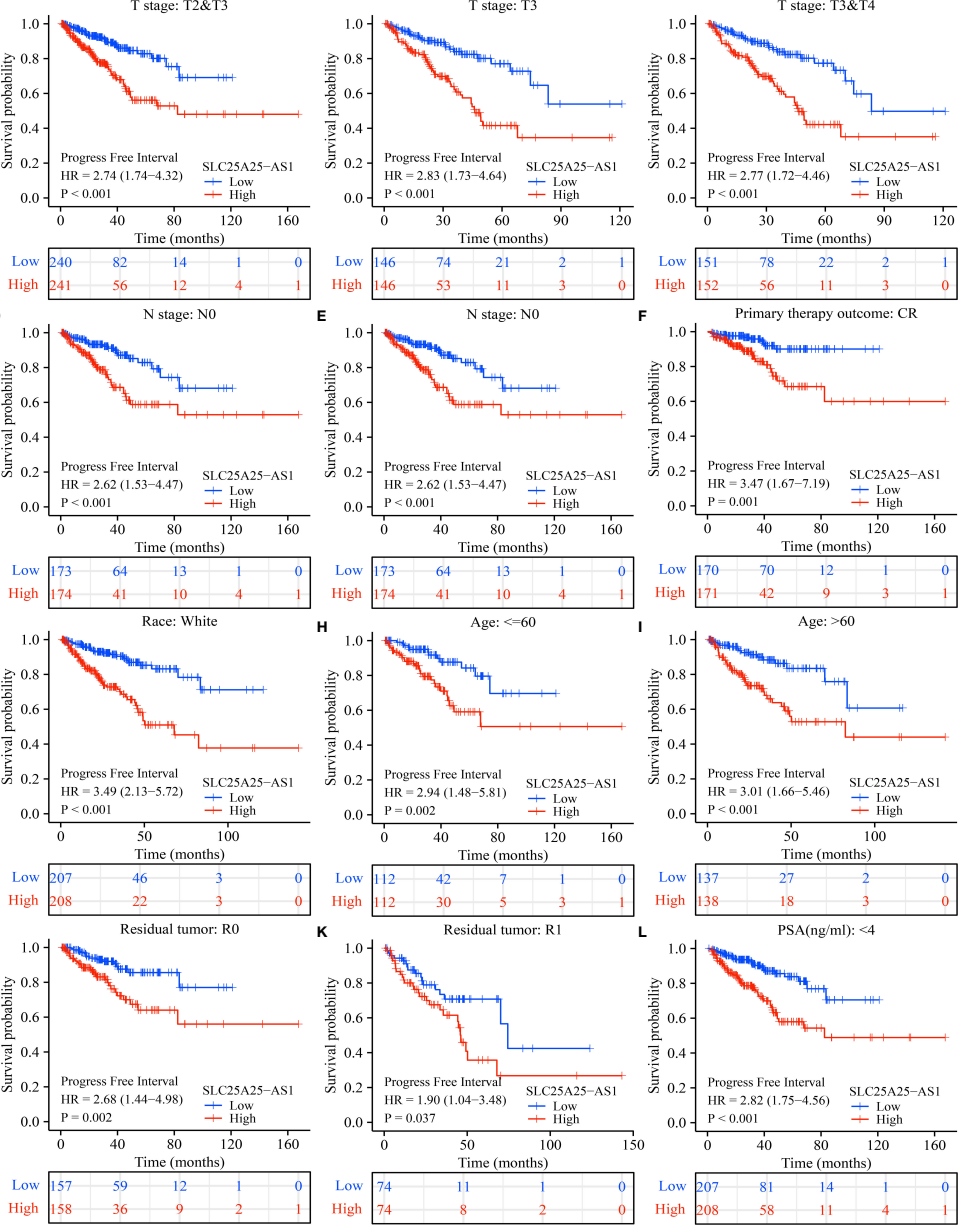
SLC25A25-AS1 overexpression was associated with the shorter PFI in subgroups of PC patients. **(A)** T2-3; **(B)** T3; **(C)** T3-4; **(D)** N0; **(E)** M0; **(F)** CR; **(G)** White; **(H)** <=60 years; **(I)** >60 years; **(J)** R0; **(K)** R1; **(L)** PSA. PC, prostate cancer; PFI, progress free interval.

COX regression analysis revealed that SLC25A25-AS1 overexpression was an independent factor for short PFI in PC patients (**Tables 1**, **2**). In addition, SLC25A25-AS1 overexpression was significantly realted to the dismal prognosis in subgroup PC patients using K-M survival. In this respect, T2-3 stage, T3 stage, T3-4 stage, N0 stage, M0 stage, CR, white people, age <=60 years, age >60 years, R0, R1, PSA (<4 ng/ml), and GS, SLC25A25-AS1 overexpression were independent predictors of the PFI in PC patients (**Figure 5** and **Figure S3**).

**Table 1:** The OS-related factors in PC patients.

Characteristics	Total (N)	HR (95% CI)	P	HR (95% CI)	P
T stage	492				
T2	189	Reference			
T3	292	3.399 (0.636-18.172)	0.153		
T4	11	0.000 (0.000-Inf)	0.998		
N stage	426				
N0	347	Reference			
N1	79	3.516 (0.778-15.896)	0.102		
M stage	458				
M0	455	Reference			
M1	3	59.383 (6.520-540.817)	<0.001	25.246 (1.452-439.073)	0.027
Primary therapy outcome	438				
PD	28	Reference			
SD	29	0.265 (0.030-2.372)	0.235	0.388 (0.036-4.248)	0.438
PR	40	0.332 (0.035-3.143)	0.336	0.325 (0.019-5.485)	0.436
CR	341	0.061 (0.011-0.340)	0.001	0.145 (0.019-1.113)	0.063
Residual tumor	468				
R0	315	Reference			
R1	148	2.635 (0.706-9.835)	0.149		
R2	5	0.000 (0.000-Inf)	0.999		
PSA (ng/ml)	442				
<4	415	Reference			
>=4	27	10.479 (2.471-44.437)	0.001	2.332 (0.321-16.949)	0.403
SLC25A25-AS1	499				
Low	249	Reference			
High	250	8.712 (1.094-69.398)	0.041	4.063 (0.453-36.417)	0.210

PC, prostate cancer; OS, overall survival.

**Table 2:** The PFI-related factors in PC patients.

Characteristics	Total (N)	HR (95% CI)	P	HR (95% CI)	P
T stage	492				
T2	189	Reference			
T3	292	3.742 (2.111-6.631)	<0.001	2.077 (1.020-4.228)	0.044
T4	11	4.879 (1.600-14.883)	0.005	4.649 (1.248-17.312)	0.022
N stage	426				
N0	347	Reference			
N1	79	1.946 (1.202-3.150)	0.007	0.959 (0.546-1.683)	0.883
M stage	458				
M0	455	Reference			
M1	3	3.566 (0.494-25.753)	0.208		
Primary therapy outcome	438				
PD	28	Reference			
SD	29	0.812 (0.400-1.650)	0.565	1.126 (0.505-2.509)	0.772
PR	40	1.528 (0.789-2.957)	0.208	1.997 (0.911-4.379)	0.084
CR	341	0.161 (0.090-0.291)	<0.001	0.308 (0.150-0.632)	0.001
Residual tumor	468				
R0	315	Reference			
R1	148	2.451 (1.623-3.699)	<0.001	0.897 (0.516-1.562)	0.702
R2	5	0.000 (0.000-Inf)	0.994	0.000 (0.000-Inf)	0.997
PSA (ng/ml)	442				
<4	415	Reference			
>=4	27	4.196 (2.095-8.405)	<0.001	1.573 (0.699-3.543)	0.274
SLC25A25-AS1	499				
Low	249	Reference			
High	250	2.905 (1.864-4.526)	<0.001	2.769 (1.615-4.750)	<0.001

PC, prostate cancer; PFI, progress free interval.

### SLC25A25-AS1 overexpression was significantly correlated with the PC immune microenvironment

**Figure 6:**
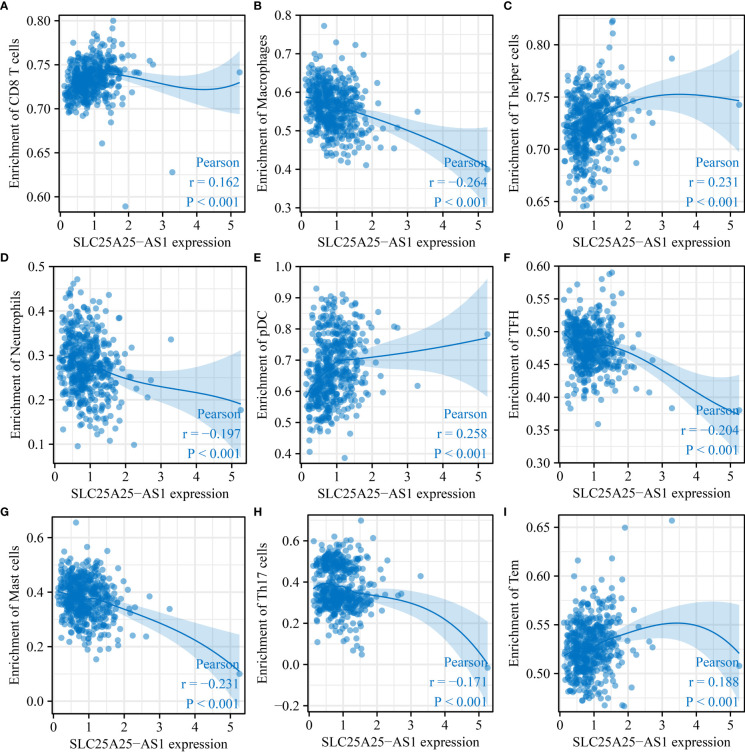
SLC25A25-AS1 overexpression was associated with immune cell infiltration in PC. **(A)** CD8 T cells; **(B)** Macrophages; **(C)** T helper cells; **(D)** pDC; **(E)** TFH; **(G)** Mast cells; **(H)** Th17 cells; **(I)** Tem. PC, prostate cancer.

**Figure 7:**
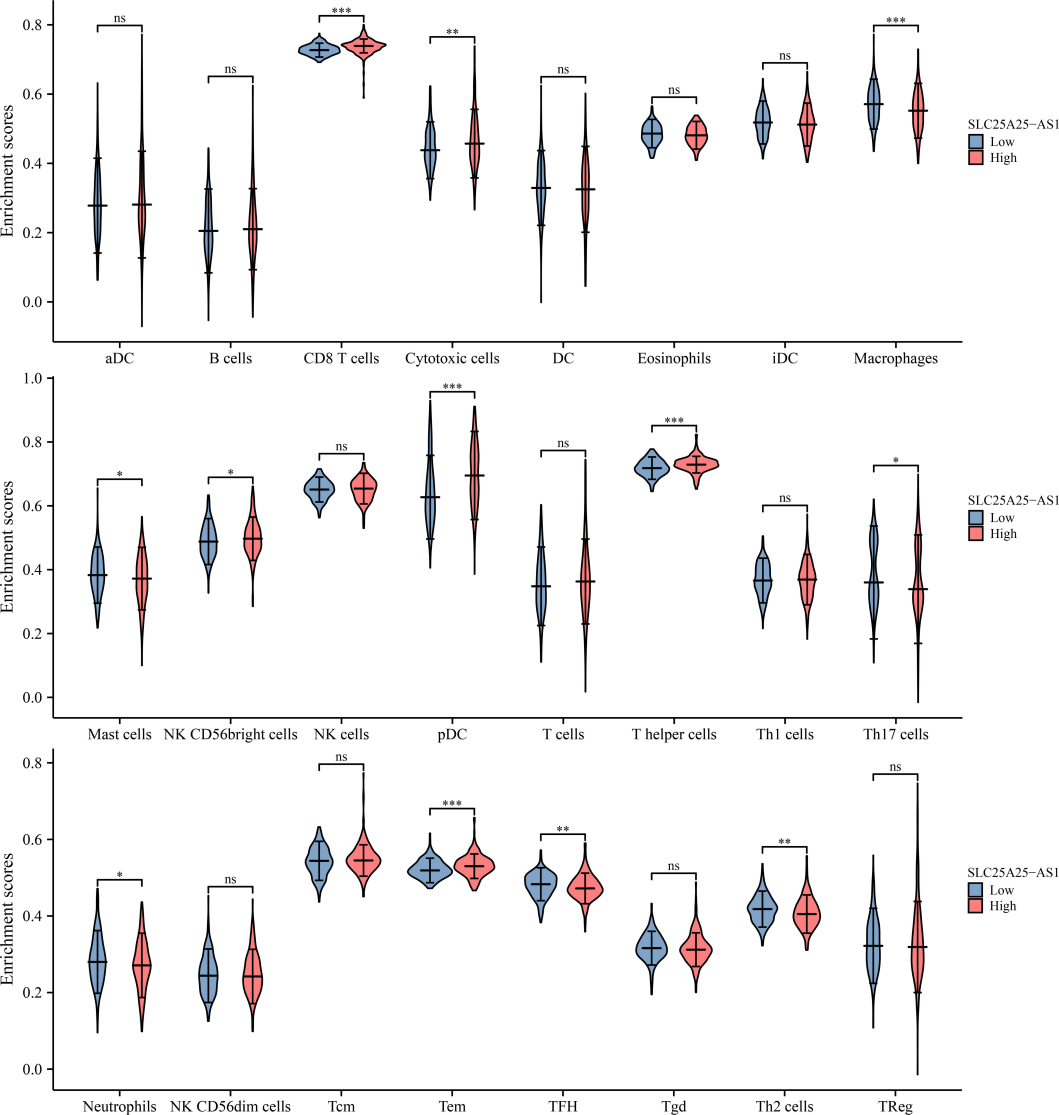
The levels of immune cells in the high- and low-expression groups of SLC25A25-AS1 ns, not statistically significant; *, P < 0.05; **,P < 0.01; ***, P < 0.001.

SLC25A25-AS1 overexpression was significantly correlated with the levels of neutrophils, CD8 T cells, macrophages, Tem, pDCs, iDCs, T helper cells, Tcm, TFH, Th17 cells, mast cells, Th2 cells, DCs, eosinophils and Th1 cells using the correlation analysis (**Figure 6** and **Figure S4**). SLC25A25-AS1 expression levels in CD8 T cells, macrophages, NK CD56bright cells, cytotoxic cells, mast cells, pDC, T helper cells, Th17 cells, neutrophils, Tem, TFH, and Th2 cells were found by grouping by the median expression value of immune cells which showed significant differences (**Figure 7**).

### Identification of SLC25A25-AS1 co-expressed lncRNAs

**Figure 8:**
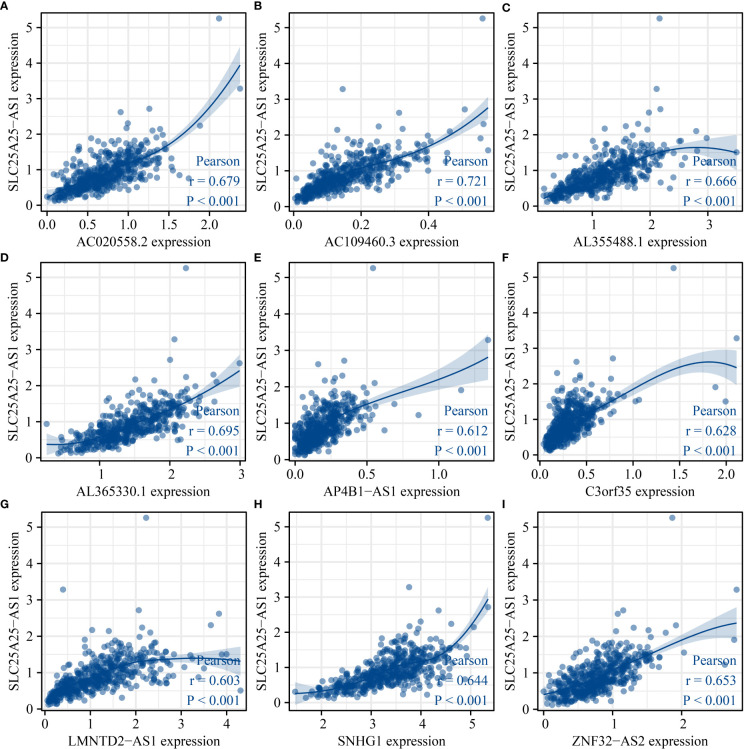
SLC25A25-AS1 related lncRNAs. **(A)** AC020558.2; **(B)** AC109460.3; **(C)** AL355488.1; **(D)** AL365330.1; **(E)** AP4B1-AS1; **(F)** C3orf35; **(G)** LMNTD2-AS1; **(H)** SNHG1; **(I)** ZNF32-AS2.

Based on the predefined screening criteria, 143 lncRNAs were co-expressed with SLC25A25-AS1 (**Table S1**), and 193 lncRNAs were significantly associated with OS in PC (P < 0.05), and 437 lncRNAs were associated with PFI (P < 0.001). The Venn plot showed 9 overlapping lncRNAs for the SLC25A25-AS1 co-expressed lncRNAs and was related to OS and PFI of PC patients, including AC020558.2, ZNF32-AS2, AP4B1-AS1, AL355488.1, AC109460.3, SNHG1, C3orf35, AL365330.1, and LMNTD2-AS1. **Figure 8** shows that SLC25A25-AS1 was correlated with AC020558.2, ZNF32-AS2, AP4B1-AS1, AL355488.1, AC109460.3, SNHG1, C3orf35, LMNTD2-AS1, and AL365330.1 expression.

### The prognostic values of SLC25A25-AS1-related lncRNAs

**Figure 9:**
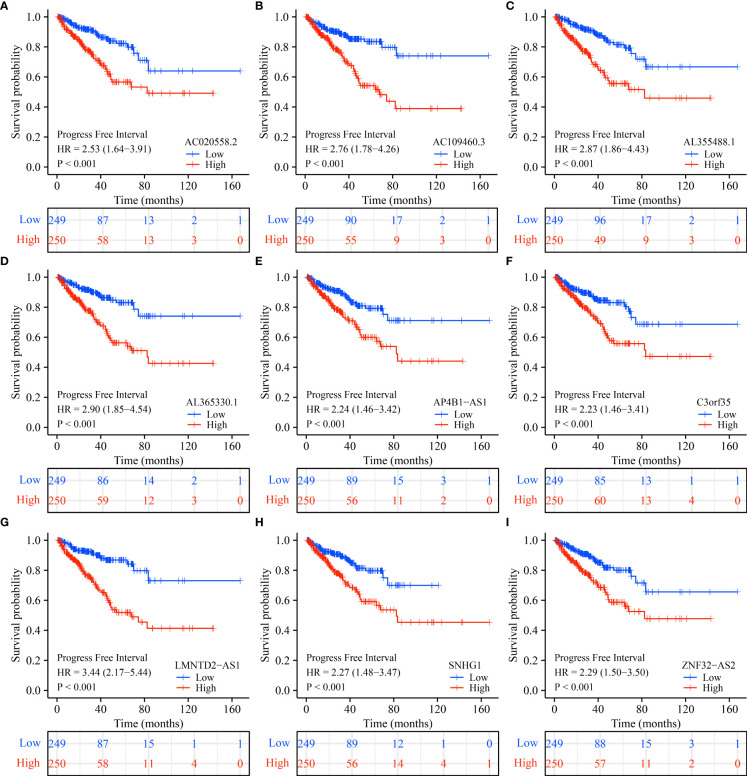
SLC25A25-AS1-related lncRNAs overexpression was associated with a shorter PFI in subgroups of PC patients. **(A)** AC020558.2; **(B)** AC109460.3; **(C)** AL355488.1; **(D)** AL365330.1; **(E)** AP4B1-AS1; **(F)** C3orf35; **(G)** LMNTD2-AS1; **(H)** SNHG1; **(I)** ZNF32-AS2. PC, prostate cancer; PFI, progress free interval.

**Figure 10:**
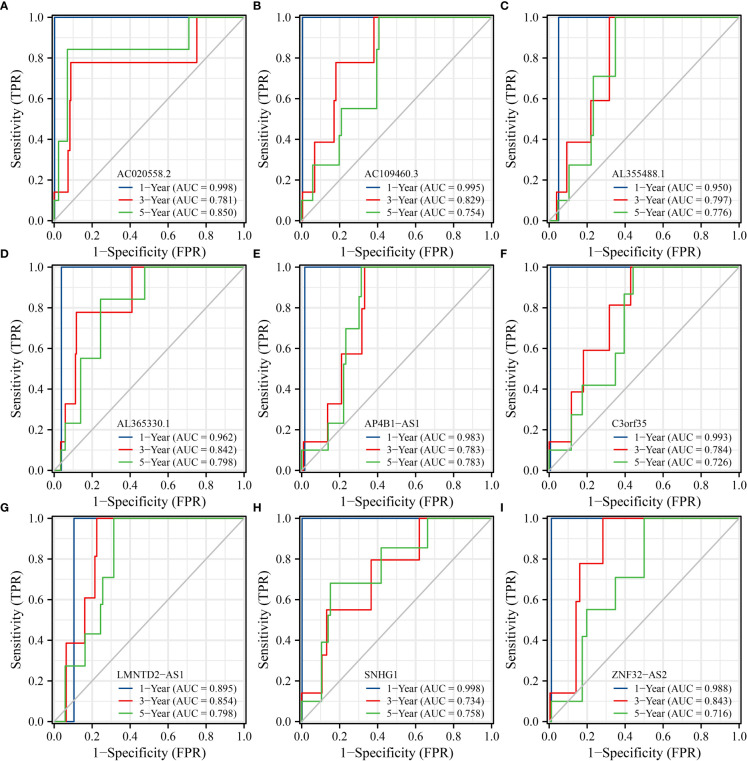
SLC25A25-AS1-related lncRNAs had significant value in predicting the OS of PC patients. **(A)** AC020558.2; **(B)** AC109460.3; **(C)** AL355488.1; **(D)** AL365330.1; **(E)** AP4B1-AS1; **(F)** C3orf35; **(G)** LMNTD2-AS1; **(H)** SNHG1; **(I)** ZNF32-AS2. PC, prostate cancer; OS, overall survival.

K-M survival analysis showed that SLC25A25-AS1-related AC020558.2, ZNF32-AS2, AP4B1-AS1, AL355488.1, AC109460.3, SNHG1, C3orf35, LMNTD2-AS1, and AL365330.1 overexpression were significantly correlated with a short OS and PFI in PC patients (**Figure S5** and **Figure 9**). ROC analysis found that overexpression of AC020558.2, ZNF32-AS2, AP4B1-AS1, AL355488.1, AC109460.3, SNHG1, C3orf35, LMNTD2-AS1, and AL365330.1 had significant value for the evaluation of the 1-, 3- and 5-year OS and PFI in PC patients (**Figure 10** and **Figure S6**).

### Construction of the risk model for SLC25A25-AS1-related lncRNAs

**Figure 11:**
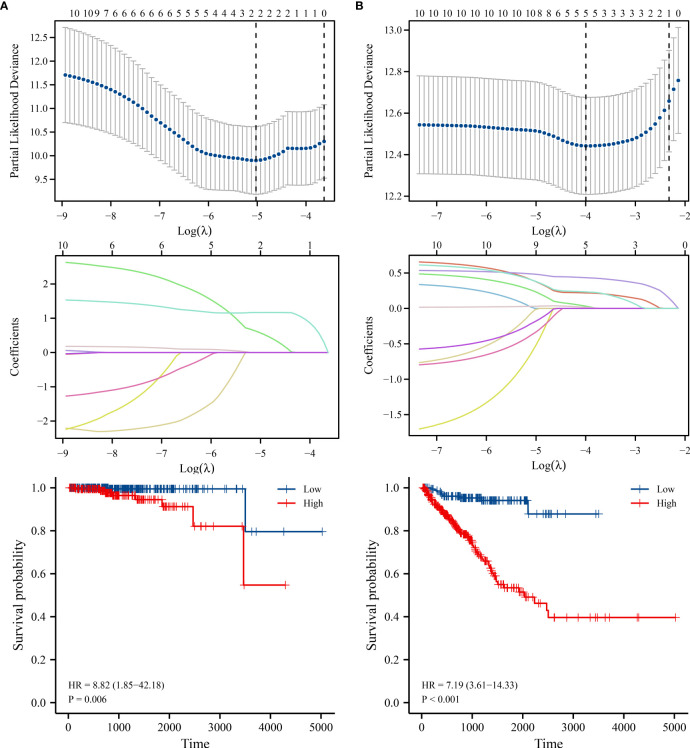
The risk models of lncRNAs in PC. **(A)** OS; **(B)** PFI. PC, prostate cancer; OS, overall survival; PFI, progress free interval.

The AC020558.2, ZNF32-AS2, AP4B1-AS1, AL355488.1, AC109460.3, SNHG1, C3orf35, LMNTD2-AS1, AL365330.1 and SLC25A25-AS1 expression data were merged with the prognosis data of PC patients. The risk factors for OS as AC020558.2 and SLC25A25-AS1 were screened by LASSO analysis. In the risk model of AC020558.2 and SLC25A25-AS1, the survival time of high-risk PC patients was significantly lower than low-risk patients (**Figure 11A**). LASSO analysis revealed that the risk factors for PFI were AC020558.2, AL355488.1, SNHG1, LMNTD2-AS1, and SLC25A25-AS1. In the risk model of AC020558.2, AL355488.1, SNHG1, LMNTD2-AS1, and SLC25A25-AS1, the PFI of high-risk PC patients was significantly lower than low-risk patients (**Figure 11B**).

## Discussion

There is a rich literature available suggesting that lncRNAs play important biological roles in PC progression (20–23). LncRNA TMPO-AS1 is increased in PC, and is relevant to a dismal prognosis. TMPO-AS1 overexpression could reduce cancer cell apoptosis and promote cell proliferation, cell cycle and migration (20). Interfering with LINC01679 expression could promote cell proliferation, metastasis, and tumor growth and inhibit cell apoptosis. Moreover, LINC01679 regulated PC cell growth and metastasis by regulating the miR-3150a-3p/SLC17A9 signaling pathway. LINC01679 and SLC17A9 expression levels were closely relevant to the dismal prognosis of PC patients (21). LOC440040 expression level was significantly upregulated in PC tissues and cells. LOC440040 overexpression in PC patients was associated with a short OS. LOC440040 overexpression was an independent risk lncRNA for dismal prognosis in PC patients. Inhibition of LOC440040 expression could inhibit cancer cell proliferation, migration and invasion (22). This finding indicated that inhibiting or promoting the expression of lncRNAs could delay cancer progression and improve the prognosis of cancer patients.

At present, overwhelming evidence substantiates that SLC25A25-AS1 plays an important role in the occurrence and development of cancer (14, 15). SLC25A25-AS1 overexpression was relevant to a dismal prognosis in patients with NSCLC. Inhibition of SLC25A25-AS1 expression could reportedly slow cancer cell growth and migration (14), while overexpression of SLC25A25-AS1 could inhibit the growth of CRC cells (15). In our study, bioinformatics and PCR analyses substantiated that SLC25A25-AS1 was overexpressed in PC tissues. TCGA database analysis showed that SLC25A25-AS1 overexpression was significantly relevant to the T stage, pathological stage, and GS in PC patients. Moreover, SLC25A25-AS1 overexpression was associated with a short OS and PFI and could predict a dismal prognosis in PC patients. SLC25A25-AS1 overexpression was significantly relevant to the OS and PFI in PC patients with T2-3 stage, T3 stage, T3-4 stage, N0 stage, M0 stage, CR, R0, R1, PSA (<4 ng/ml), and GS. Our preliminary findings suggest that SLC25A25-AS1 functions as an oncogene in PC progression and is expected to be a future biomarker for this patient population.

The risk models have been used to assess the dismal prognosis of cancer patients (24–28). The expression levels of AC012085.2, UBXN10-AS1 and LINC00261 were significantly downregulated, while the AP004608.1, AC104667.2, and AC008610.1 expression levels were upregulated in PC tissues. The risk model based on AP004608.1, LINC00261, AC012085.2, AC104667.2, UBXN10-AS1, and AC008610.1 was applied to evaluate the dismal prognosis of PC patients. In addition, a lncRNA-miRNA-mRNA regulatory network was established based on 6 autophagy-related AC008610.1, LINC00261, AC012085.2, AP004608.1, UBXN10-AS1, and AC104667.2, 17 miRNAs, and 12 autophagy genes to understand the signaling mechanisms underlying PC progression (25). NOL12 expression is reportedly upregulated in hepatocellular carcinoma (HCC) tissues and cells and correlates with a short OS. Inhibition of NOL12 expression levels could inhibit HCC cell growth and metastasis. The risk model based on the NOL12-related genes was an independent prognostic factor in patients with HCC. The survival of HCC patients in the low-risk group was significantly better than in the high-risk group (28). We found SLC25A25-AS1-related AC020558.2, ZNF32-AS2, AP4B1-AS1, AL355488.1, AC109460.3, SNHG1, C3orf35, LMNTD2-AS1, and AL365330.1 overexpression levels were associated with a short OS, and PFI, and exhibited significant value in diagnosing 1-, 3-, and 5-year suvival time in PC patients. In the risk model constructed, the OS and PFI of high-risk PC patients were significantly lower than low-risk patients, which indicated that our constructed risk model has huge prospects for application in evaluating the prognosis of PC patients.

Immunotherapy has attracted significant interest in recent years for cancer treatment (12, 29–32). Programmed cell death 1/programmed cell death ligand 1 (PD-1/PDL-1) and immune checkpoint inhibitors targeting PD-1/PD-L1 play an important role in the treatment of pan-cancer patients. For example, PD-L1 overexpression was associated with dismal clinical prognosis in PC patients. The downregulation of PD-L1 expression levels in PC yields an anti-tumor effect (31). We found that SLC25A25-AS1 overexpression was significantly relevant to the PC immune microenvironment. Moreover, SLC25A25-AS1 overexpression was significantly relevant to the CD8 T cells, macrophages, Tcm, and other immune cell levels. Overall, we preliminarily demonstrated that the SLC25A25-AS1 expression level was relevant to the PC immune microenvironment.

In this study, the roles of SLC25A25-AS1 in PC progression were comprehensively analyzed, indicating that SLC25A25-AS1 has huge prospects as a prognostic biomarker for PC patients. However, the clinical values of SLC25A25-AS1 and the risk model warrant further validation in clinical samples and analysis of the roles and signaling mechanisms of SLC25A25-AS1 in PC cell growth, migration, and tumorigenicity should be conducted in xenograft models. Future studies should focus on inhibition of the expression of SLC25A25-AS1 in PC cells and establishing xenograft models. In addition, the roles of SLC25A25-AS1 expression in PC immune cell infiltration should be investigated *in vitro* and *in vivo*. Taken together, our findings corroborate that the expression of SLC25A25-AS1 is significantly increased in PC tissues and associated with clinicopathological features and dismal prognosis. SLC25A25-AS1 overexpression was significantly relevant to the infiltration of CD8 T cells, iDCs, macrophages, etc. Besides, AC020558.2, ZNF32-AS2, AP4B1-AS1, AL355488.1, AC109460.3, SNHG1, C3orf35, LMNTD2-AS1, and AL365330.1 were significantly associated with the OS and PFI in PC patients. The risk models based on SLC25A25-AS1-related lncRNAs were relevant to a dismal prognosis and are expected to become prognostic tools for this patient population.

## Data availability statement

The original contributions presented in the study are included in the article/**Supplementary Material**. Further inquiries can be directed to the corresponding authors.

## Ethics statement

The studies involving human participants were reviewed and approved by The Ethics Committee of the Affiliated Hospital of Zunyi Medical University. The patients/participants provided their written informed consent to participate in this study.

## Author contributions

K-WY and R-SS established the central thrust of our study. Y-YZ and Q-MX drafted the manuscript, which was revised by DK, and J-LC. LZ and W-LZ analyzed the research data. All authors contributed to the article and approved the submitted version.

## Funding

Our study was funded by the Zunyi City Joint Fund (Zun Shi Ke He HZ Word (2021) No. 73, and Zun Shi Ke He HZ Word (2022) No. 244).

## Acknowledgments

We are extremely grateful for the support and help from ourdepartment at the Affiliated Hospital of Zunyi Medical University.

## Conflict of interest

The authors declare that the research was conducted in the absence of any commercial or financial relationships that could be construed as a potential conflict of interest.

## Publisher’s note

All claims expressed in this article are solely those of the authors and do not necessarily represent those of their affiliated organizations, or those of the publisher, the editors and the reviewers. Any product that may be evaluated in this article, or claim that may be made by its manufacturer, is not guaranteed or endorsed by the publisher.
