# Supplementary material for: SLC25A25-AS1 over-expression could be predicted the dismal prognosis and was related to the immune microenvironment in prostate cancer
Source: Front Oncol. 2022 Oct 20;12:990247. doi: 10.3389/fonc.2022.990247 (PMC9632290; doi:10.3389/fonc.2022.990247)
Supplement: Supplementary file 1 [file DataSheet_1.docx]

***Supplementary Materials***

Supplementary Figures


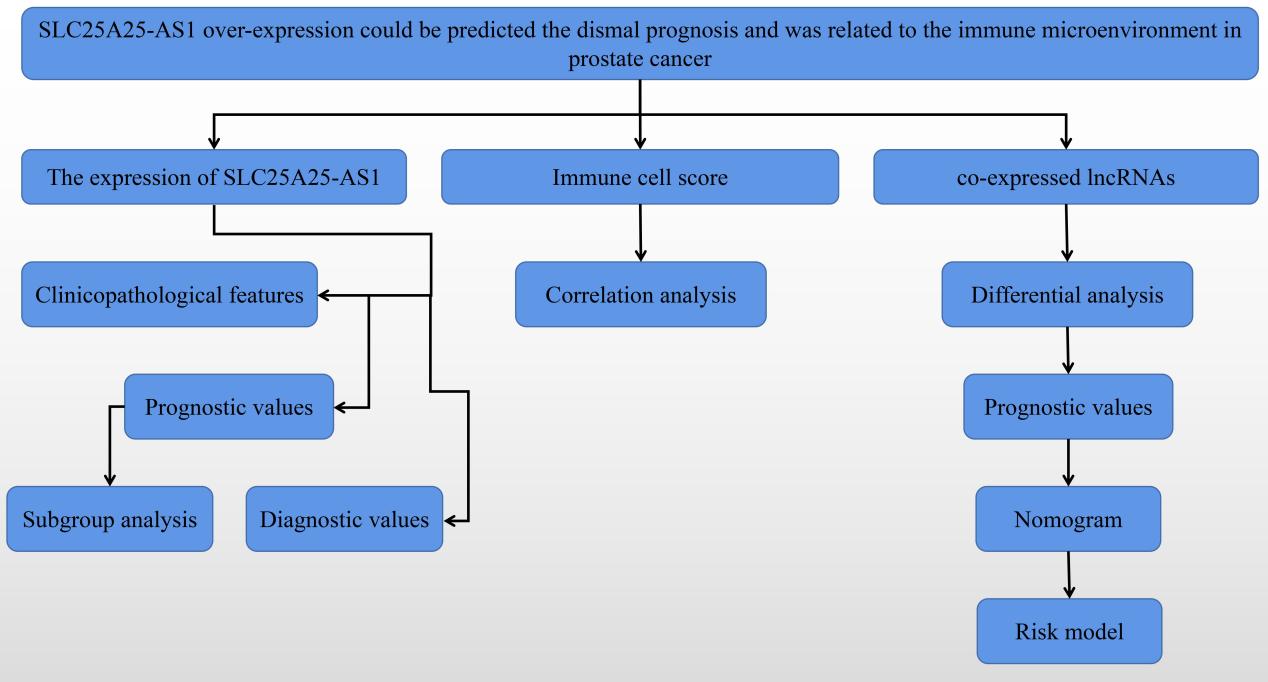


Figure S1. Graphical abstract of this study.


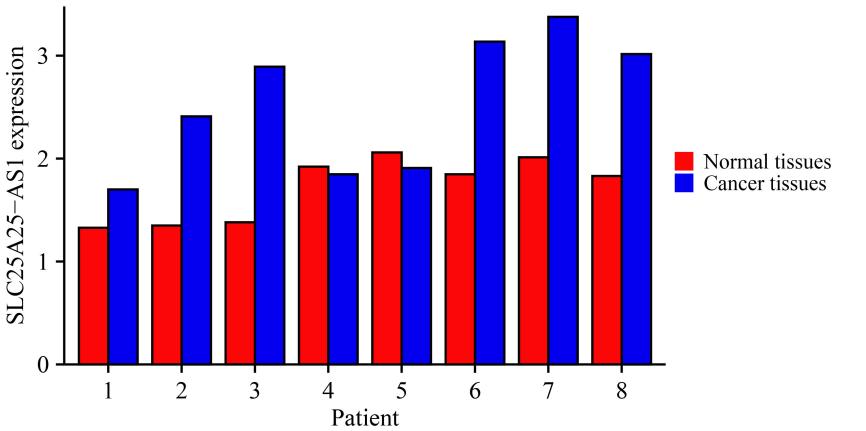


Figure S2. The expression level of SLC25A25-AS1 in 8 PC patients.


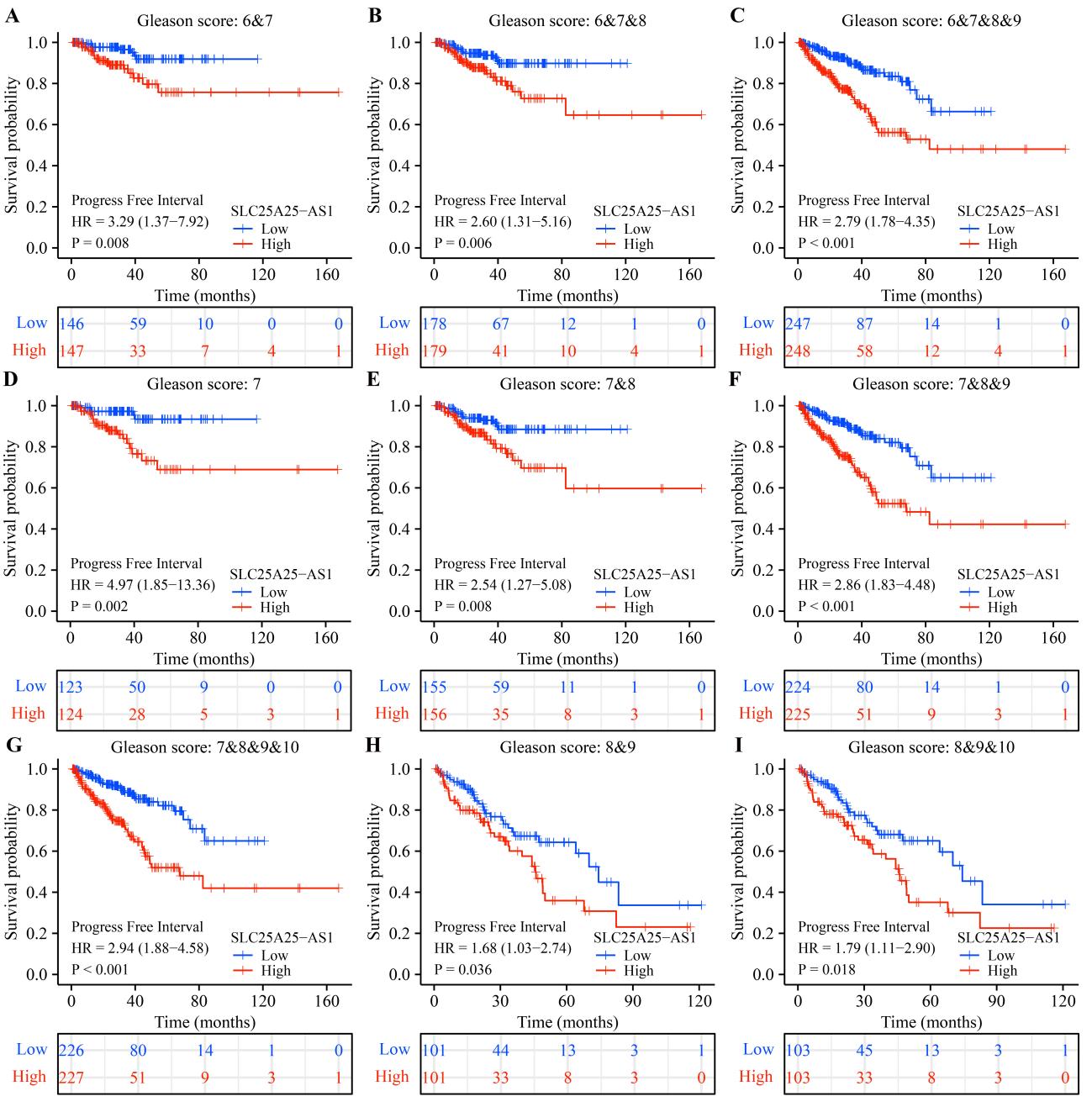


Figure S3. SLC25A25-AS1 overexpression was associated with a shorter PFI in the GS subgroups.

Note: GS, Gleason score; PFI, progress free interval.


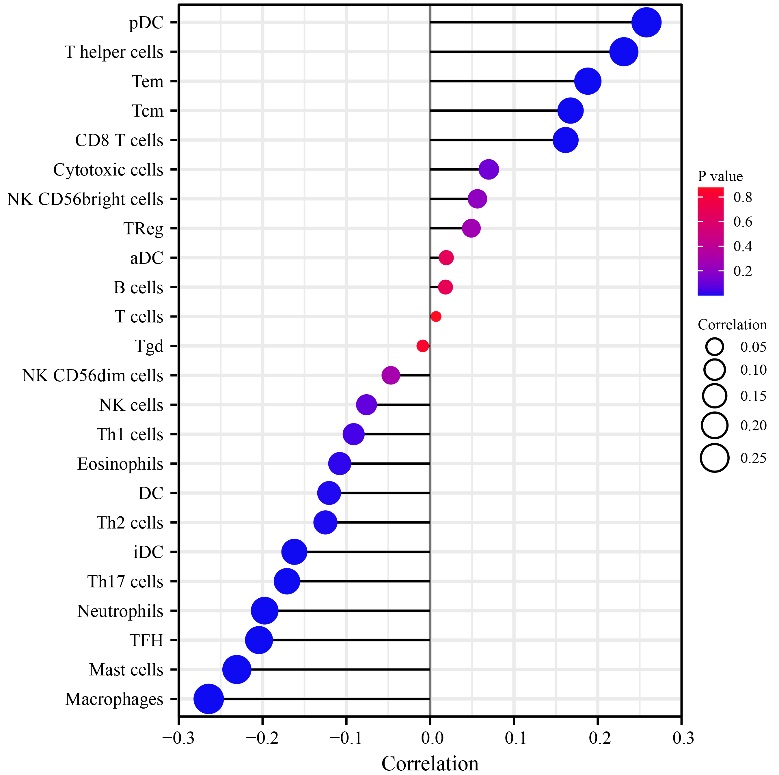


Figure S4. SLC25A25-AS1 overexpression was relevant to the immune cells in PC.

Note: PC, prostate cancer


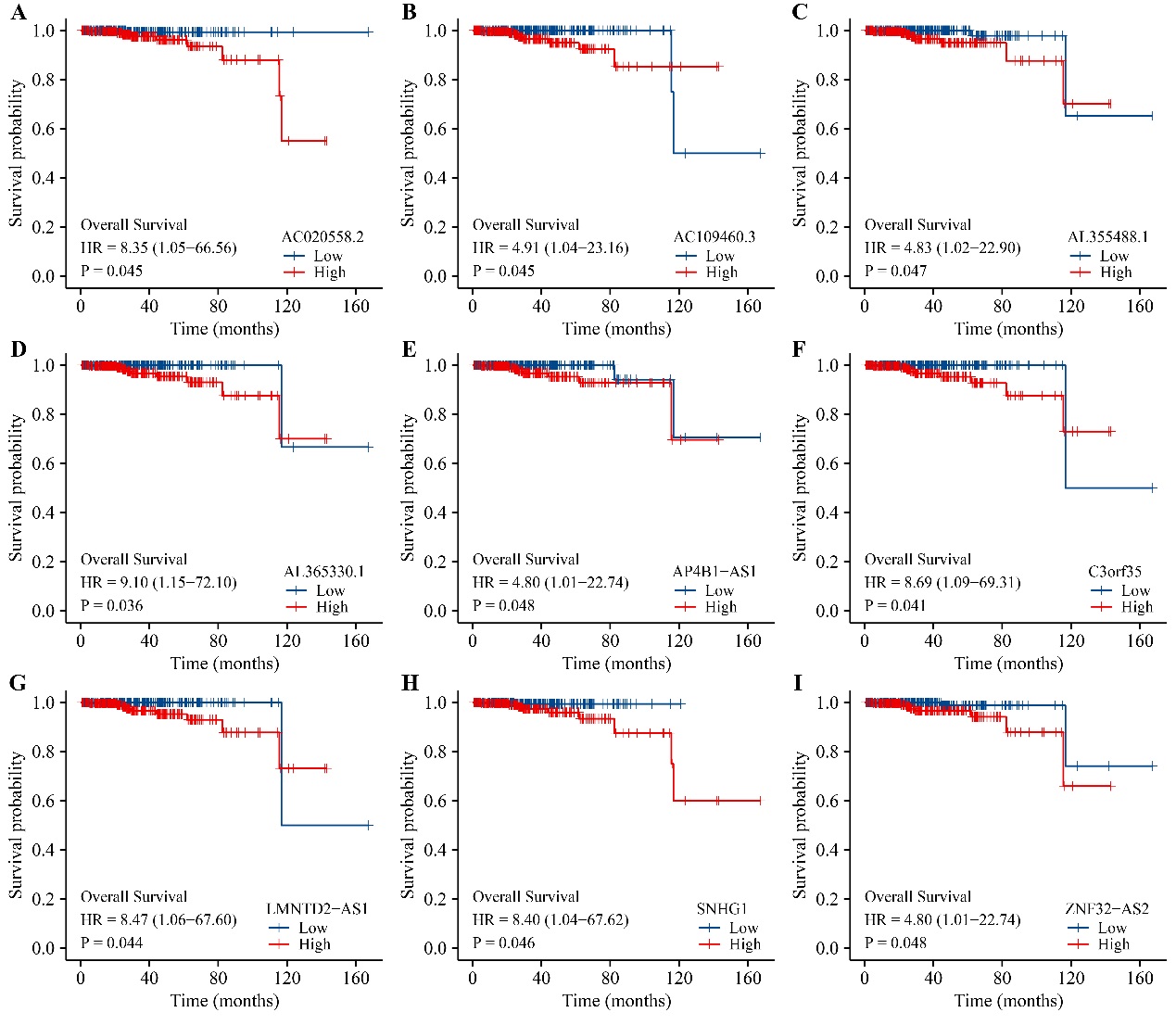


Figure S5. SLC25A25-AS1-related lncRNAs overexpression was associated with a shorter OS in the subgroup of PC patients. (A) AC020558.2; (B) AC109460.3; (C) AL355488.1; (D) AL365330.1; (E) AP4B1-AS1; (F) C3orf35; (G) LMNTD2-AS1; (H) SNHG1; (I) ZNF32-AS2.

Note: PC, prostate cancer; OS, overall survival.


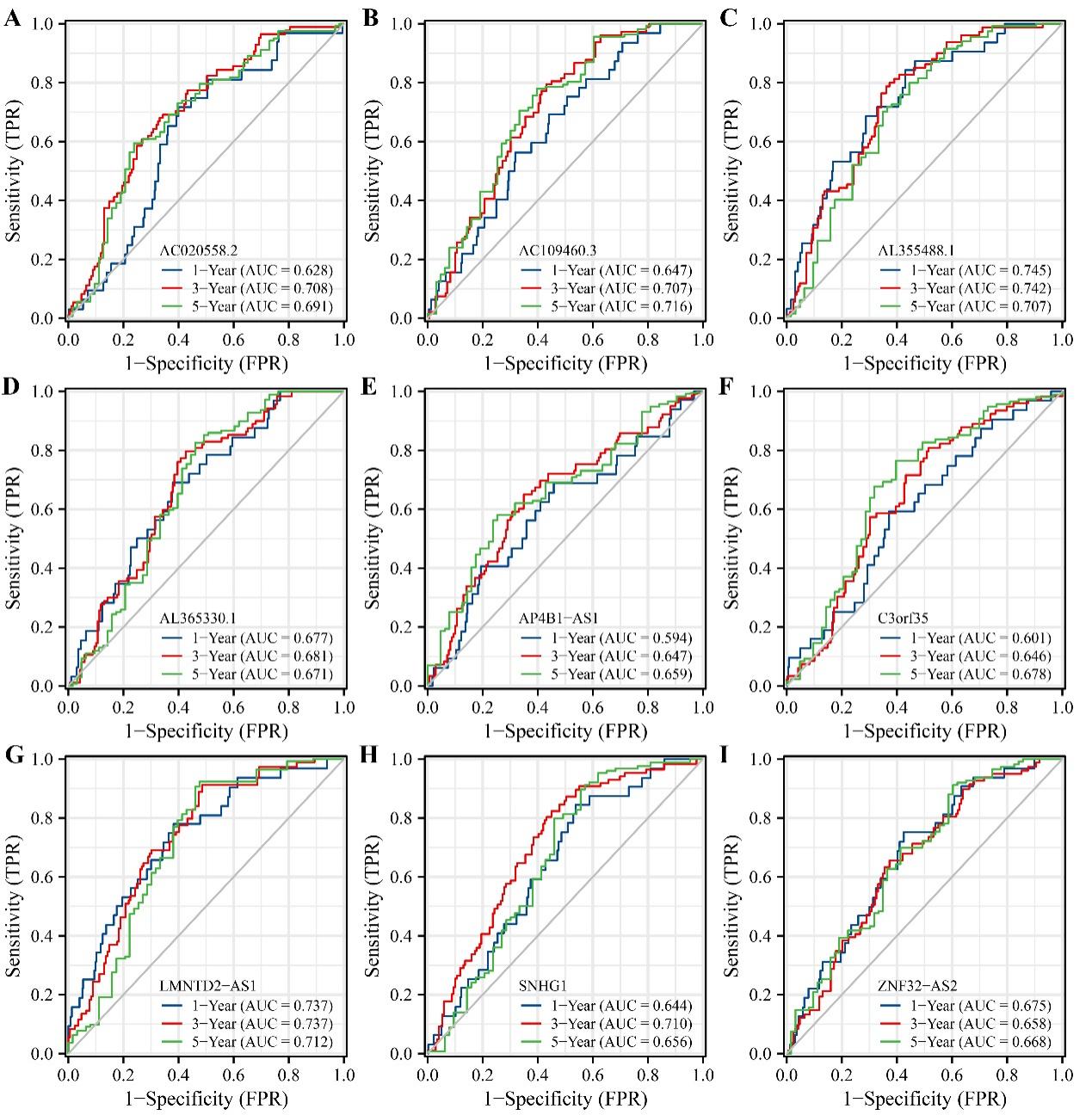


Figure S6. SLC25A25-AS1-related lncRNAs had significant value in predicting the PFI of PC patients. (A) AC020558.2; (B) AC109460.3; (C) AL355488.1; (D) AL365330.1; (E) AP4B1-AS1; (F) C3orf35; (G) LMNTD2-AS1; (H) SNHG1; (I) ZNF32-AS2.

Note: PC, prostate cancer; PFI, progress free interval.

Supplementary Tables

Table S1. SLC25A25-AS1 co-expressed lncRNAs.

| LncRNA | r | P |
| --- | --- | --- |
| Z97832.2 | 0.643473636 | 1.1366E-59 |
| AC002128.1 | 0.652411666 | 7.85129E-62 |
| AC092611.1 | 0.621250577 | 1.34008E-54 |
| AC073957.3 | 0.700832597 | 6.182E-75 |
| AC040162.3 | 0.702692794 | 1.71716E-75 |
| Z84485.1 | 0.649047512 | 5.20871E-61 |
| AC026362.1 | 0.652143621 | 9.13698E-62 |
| C3orf35 | 0.628390775 | 3.4874E-56 |
| PCBP1-AS1 | 0.652796769 | 6.31248E-62 |
| AC027796.4 | 0.638502911 | 1.68385E-58 |
| AL109811.2 | 0.6637809 | 1.09263E-64 |
| AC234582.1 | 0.611852547 | 1.41737E-52 |
| AL117209.1 | 0.614708941 | 3.49481E-53 |
| RNF139-AS1 | 0.651612213 | 1.23361E-61 |
| AC018766.1 | 0.60875771 | 6.35824E-52 |
| MUC20-OT1 | 0.651778878 | 1.12284E-61 |
| SH3BP5-AS1 | 0.669884899 | 2.83423E-66 |
| AC008870.2 | 0.652925269 | 5.86889E-62 |
| AC011481.1 | 0.615050467 | 2.95327E-53 |
| AC055855.1 | 0.604143226 | 5.78144E-51 |
| AC009120.2 | 0.66106567 | 5.39538E-64 |
| KLF3-AS1 | 0.601495843 | 2.01845E-50 |
| AC021739.2 | 0.649161519 | 4.88711E-61 |
| AP001107.4 | 0.698183863 | 3.76586E-74 |
| INE1 | 0.622626778 | 6.68185E-55 |
| AL139349.1 | 0.646223876 | 2.50313E-60 |
| MED8-AS1 | 0.643084957 | 1.40582E-59 |
| LMNTD2-AS1 | 0.602557725 | 1.22415E-50 |
| AL031714.1 | 0.662945574 | 1.78901E-64 |
| MIR600HG | 0.600210791 | 3.68774E-50 |
| AC105020.1 | 0.623641117 | 3.99178E-55 |
| LINC02883 | 0.6038457 | 6.65753E-51 |
| LINC00894 | 0.632322511 | 4.48902E-57 |
| AC004148.1 | 0.733133703 | 2.96057E-85 |
| AC129510.1 | 0.638019809 | 2.18239E-58 |
| AL355488.1 | 0.665570079 | 3.7798E-65 |
| PTOV1-AS2 | 0.626625709 | 8.67086E-56 |
| PSMA3-AS1 | 0.629056689 | 2.46947E-56 |
| AL161452.1 | 0.654289968 | 2.70108E-62 |
| AC008735.2 | 0.756362996 | 1.1655E-93 |
| SEMA3F-AS1 | 0.685191433 | 2.01213E-70 |
| SLC9A3-AS1 | 0.638751009 | 1.47361E-58 |
| AF111169.3 | 0.644865664 | 5.29481E-60 |
| ZKSCAN2-DT | 0.659013376 | 1.78422E-63 |
| LINC00106 | 0.623786242 | 3.70757E-55 |
| GUSBP11 | 0.646788999 | 1.83069E-60 |
| AC020907.4 | 0.640142748 | 6.95794E-59 |
| AC012360.3 | 0.63624311 | 5.64191E-58 |
| ZNF32-AS2 | 0.653458385 | 4.33619E-62 |
| AC005332.5 | 0.641119545 | 4.09956E-59 |
| AC137932.3 | 0.631013844 | 8.91101E-57 |
| AC020558.2 | 0.679099851 | 9.66139E-69 |
| AL162586.1 | 0.749635553 | 3.94276E-91 |
| AC006435.2 | 0.67683693 | 3.97472E-68 |
| THUMPD3-AS1 | 0.63640031 | 5.18846E-58 |
| AL139287.1 | 0.657821086 | 3.55911E-63 |
| AP4B1-AS1 | 0.611800104 | 1.45408E-52 |
| CR559946.2 | 0.600592571 | 3.08406E-50 |
| SPAG5-AS1 | 0.601283188 | 2.23056E-50 |
| ERVK13-1 | 0.603285909 | 8.6782E-51 |
| AC130650.2 | 0.603290226 | 8.6605E-51 |
| GARS1-DT | 0.681119611 | 2.7045E-69 |
| SLFNL1-AS1 | 0.643821389 | 9.39484E-60 |
| AC024361.3 | 0.618186681 | 6.23207E-54 |
| LINC01355 | 0.697053898 | 8.09134E-74 |
| STAG3L5P-PVRIG2P-PILRB | 0.648939898 | 5.53154E-61 |
| AC011462.4 | 0.664534074 | 6.99515E-65 |
| SNHG12 | 0.61793488 | 7.06582E-54 |
| AC090589.3 | 0.671165338 | 1.30291E-66 |
| AC232271.1 | 0.612770515 | 9.05204E-53 |
| AL512770.1 | 0.695661731 | 2.06597E-73 |
| MMP25-AS1 | 0.719852575 | 7.77395E-81 |
| LINC00174 | 0.633994665 | 1.86028E-57 |
| AC073575.2 | 0.612971618 | 8.20351E-53 |
| AL928654.2 | 0.651595923 | 1.245E-61 |
| AC008735.1 | 0.665368217 | 4.26227E-65 |
| LINC01004 | 0.630387498 | 1.23578E-56 |
| GHRLOS | 0.641532871 | 3.27545E-59 |
| AC027601.1 | 0.696852691 | 9.26837E-74 |
| AC110285.2 | 0.610330181 | 2.97189E-52 |
| AP006623.1 | 0.655315284 | 1.50376E-62 |
| AC011005.4 | 0.619702756 | 2.91919E-54 |
| AC245060.6 | 0.626379535 | 9.84076E-56 |
| LINC00342 | 0.651405038 | 1.38655E-61 |
| AC008735.4 | 0.713733579 | 6.94275E-79 |
| AL022328.2 | 0.686568601 | 8.27555E-71 |
| AC007566.1 | 0.64959923 | 3.82536E-61 |
| POLR2J4 | 0.619291714 | 3.58707E-54 |
| LENG8-AS1 | 0.713955131 | 5.91278E-79 |
| RUSC1-AS1 | 0.690348849 | 7.039E-72 |
| AL109811.1 | 0.635023956 | 1.07873E-57 |
| AC092119.2 | 0.678870689 | 1.11557E-68 |
| AP001062.1 | 0.621365993 | 1.26427E-54 |
| AP006621.2 | 0.68518512 | 2.02032E-70 |
| AC004076.2 | 0.647912581 | 9.80899E-61 |
| AL583810.2 | 0.637297947 | 3.21258E-58 |
| AC069222.1 | 0.613921169 | 5.14924E-53 |
| AC024361.1 | 0.621764202 | 1.03397E-54 |
| TMEM147-AS1 | 0.646069354 | 2.72636E-60 |
| AC087289.2 | 0.611136518 | 2.00881E-52 |
| AL391684.1 | 0.633809126 | 2.05184E-57 |
| AP002907.1 | 0.605215282 | 3.473E-51 |
| AC010201.2 | 0.680379902 | 4.31634E-69 |
| MZF1-AS1 | 0.632812408 | 3.46985E-57 |
| AL161729.4 | 0.70349121 | 9.87875E-76 |
| AC012615.6 | 0.714056654 | 5.49303E-79 |
| AC005519.1 | 0.631862452 | 5.71474E-57 |
| AC048341.1 | 0.656483299 | 7.69494E-63 |
| AC015813.1 | 0.624203449 | 2.99764E-55 |
| SNHG1 | 0.64365275 | 1.03042E-59 |
| AC010618.3 | 0.641853873 | 2.75088E-59 |
| AL359715.1 | 0.604943684 | 3.95237E-51 |
| AC114730.3 | 0.658281576 | 2.72696E-63 |
| AC010973.2 | 0.679065102 | 9.87447E-69 |
| AC002553.1 | 0.696289193 | 1.35497E-73 |
| CACTIN-AS1 | 0.6386058 | 1.59327E-58 |
| AC127024.5 | 0.622037619 | 9.00469E-55 |
| AL049795.1 | 0.614248293 | 4.38435E-53 |
| AL121832.3 | 0.603629606 | 7.37527E-51 |
| AC093495.1 | 0.60019498 | 3.71513E-50 |
| AC084018.1 | 0.648439898 | 7.3122E-61 |
| CAPN10-DT | 0.620969833 | 1.54384E-54 |
| AL583810.1 | 0.63209436 | 5.0602E-57 |
| AL022328.1 | 0.664116513 | 8.9586E-65 |
| AC132872.3 | 0.668218475 | 7.74776E-66 |
| AC004253.1 | 0.662629876 | 2.15459E-64 |
| AL136295.2 | 0.602806156 | 1.0887E-50 |
| AC009404.1 | 0.691611709 | 3.06394E-72 |
| PRKCZ-AS1 | 0.648410456 | 7.43323E-61 |
| AL135999.1 | 0.679096079 | 9.6843E-69 |
| AC109460.3 | 0.720644335 | 4.30919E-81 |
| AL365330.1 | 0.694941723 | 3.34779E-73 |
| AC005306.1 | 0.628662284 | 3.02991E-56 |
| AC007038.1 | 0.634437466 | 1.47189E-57 |
| AL592211.1 | 0.697698171 | 5.23389E-74 |
| AC084125.2 | 0.636287762 | 5.50925E-58 |
| AC021739.4 | 0.626802788 | 7.91576E-56 |
| ZFHX2-AS1 | 0.646913038 | 1.70905E-60 |
| AC245884.8 | 0.641489785 | 3.35303E-59 |
| AC097641.2 | 0.615281053 | 2.63563E-53 |
| ZNF32-AS1 | 0.613527349 | 6.24757E-53 |
| AC005253.1 | 0.648175448 | 8.47343E-61 |
| AP001628.1 | 0.659200765 | 1.60026E-63 |
